# Fractures and Dislocations of the Carpal Bones

**Published:** 1904-10-08

**Authors:** 


### FRACTURES AND DISLOCATIONS OF THE CARPAL BONES.

As a consequence of the increasing use of skiagraphy for diagnostic purposes, it is becoming daily more apparent that many injuries which have formerly been regarded as instances of contused bone, or have been included under the vague term "sprain," are in reality cases of fracture. Thus numerous examples could be quoted of complete transverse fractures of the long bones, where the usual and manifest signs of fracture have been absent, owing, presumably, to the integrity of the periosteum; and many of the so-called "sprains" about the hip, ankle, and wrist are now found to be associated with broken bones. It is important to urge this fact upon the profession; indeed, it may be enunciated as one of the guiding principles in medical practice to submit every case of "sprain" or "contused bone" to a competent skiagrapher whenever such a course is possible, both as a useful help towards diagnosis, and as a measure of protection to the medical attendant. The grave injury that may be inflicted on the doctor, especially in country districts and small towns, by an erstwhile patient whom he has treated for a "sprain," and who, because of prolonged pain and disability of the joint, subsequently discovers through an independent *x*-ray examination that a fracture is present, can scarcely be exaggerated.

After these preliminary remarks we may usefully draw attention to a paper by Dr. W. S. Haughton and Major M. P. Holt,[1] R.A.M.C. These writers have observed a number of cases of injuries to the wrist where the *x* rays have revealed fracture or dislocation of one or more of the carpal bones of the proximal row; and their experience has led them to put forward the proposition that, with a history of recent injury to the wrist followed by extreme pain on attempted movement, but with possibly no deformity, or with a history of old injury followed by more or less complete and permanent impairment of function, there will be found in almost all cases either fracture or more or less complete dislocation of one or more of the carpal bones

The violence necessary to produce these injuries is not necessarily extreme, and it varies greatly in form. Among the 10 cases which they record there was fracture of the scaphoid bone in eight, and there are some features connected with this injury that seem to be fairly characteristic. The injured hand is held in a position of extension and adduction, with marked resistance to attempts to abduct or flex the hand, while the point of greatest tenderness is over the scaphoid. The fracture is most commonly a transverse one through the middle of the bone, where the circumference is smallest.

Treatment, in cases of simple fracture without displacement, consists of massage and movement; and these should be repeated daily from the first. When a fragment is displaced it should be removed. When one or more bones are dislocated and cannot be reduced, excision through a dorsal incision is indicated, and a useful joint will result. With regard to the taking of the *x* ray photographs, the authors lay stress on the value of stereoscopic skiagrams, and advise that in difficult cases, for purposes of comparison, the two wrists should be photographed in the following positions: (1) both prone, (2) both fully supinated, (3) both semi-prone; and they add that it is absolutely necessary, for all comparative work, that some definite and constant points should be invariably made use of over which to place the anti-kathode.

[1] Jour. R.A.M.C., September, 1904.

